# From External to Local: Opportunities and Lessons Learned from Transitioning COMSA-Mozambique

**DOI:** 10.4269/ajtmh.22-0284

**Published:** 2023-04-10

**Authors:** Daniela C. Rodríguez, Ivalda Macicame, Antonio Adriano, Sergio Chicumbe, Pedro Duce, Almamy Kante, Victor A. Mavie, Etelvina Mbalane, Sheila Nhachungue, Nordino Titus, Fred Van Dyk, Agbessi Amouzou

**Affiliations:** 1 Department of International Health, Johns Hopkins Bloomberg School of Public Health, Baltimore, Maryland;; 2 Instituto Nacional de Saúde, Maputo, Mozambique;; 3 Instituto Nacional de Estatística, Maputo, Mozambique;; 4 Independent Consultant, Maputo, Mozambique

## Abstract

Donor transitions, where externally funded programs transfer to country ownership and management, are increasingly common. The Countrywide Mortality Surveillance for Action – Mozambique (COMSA) project established a nationwide surveillance system capturing vital events at the community level with funding from the Bill and Melinda Gates Foundation. COMSA was implemented in partnership between Johns Hopkins University (a U.S.-based academic institution) and the Instituto Nacional de Saúde (National Institute for Health) and Instituto Nacional de Estatística (National Institute for Statistics), two Mozambican public institutions. Midway through the project, the Gates Foundation directed COMSA’s partners to develop and implement a transition plan that ensured COMSA’s activities could be institutionalized after Gates Foundation funding ended. Here we describe the process and activities that COMSA underwent for transition planning, including stakeholder engagement and advocacy, securing financial commitments, documenting operational activities, capacity building, and supporting strategic planning. Facilitators included a project model that already embedded significant implementation and management responsibility with local agencies, high-level commitment to COMSA’s activities from local stakeholders, establishing dedicated personnel and budget to manage transition, and fortuitous timing for financing. Challenges included needing to engage multiple government agencies to ensure buy-in, navigating tensions around future roles and responsibilities, reviewing and adjusting existing implementation structures, and the reality that this transition involved shifting financing from one development partner to another. Transition implementation was also constrained by the COVID-19 pandemic because key stakeholders were engaged in response efforts. COMSA’s experience highlights lessons and threats for future programs facing donor transition in uncertain environments.

## INTRODUCTION

Donor transitions, whereby programs that have been developed, financed, and supported by development partners are transferred to local stakeholders (government and non-government), are becoming increasingly common in the global health development assistance landscape.^1^ Donor transitions in low- and middle-income countries and their impacts have been documented in family planning,^2^^,^^3^ immunization,^4^^,^^5^ and HIV/AIDS.^6^^–^^9^ Several funders have proactively monitored and evaluated their transitions with a learning objective for themselves and others,^10^^–^^12^ and others, like Gavi and The Global Fund, developed explicit transition policies to prepare country counterparts for taking increased responsibility for program management and financing.^13^^,^^14^ Recent studies have highlighted the lack of consistency between transition approaches^15^ as well as concerns from local policymakers.^16^

**Figure 1.**
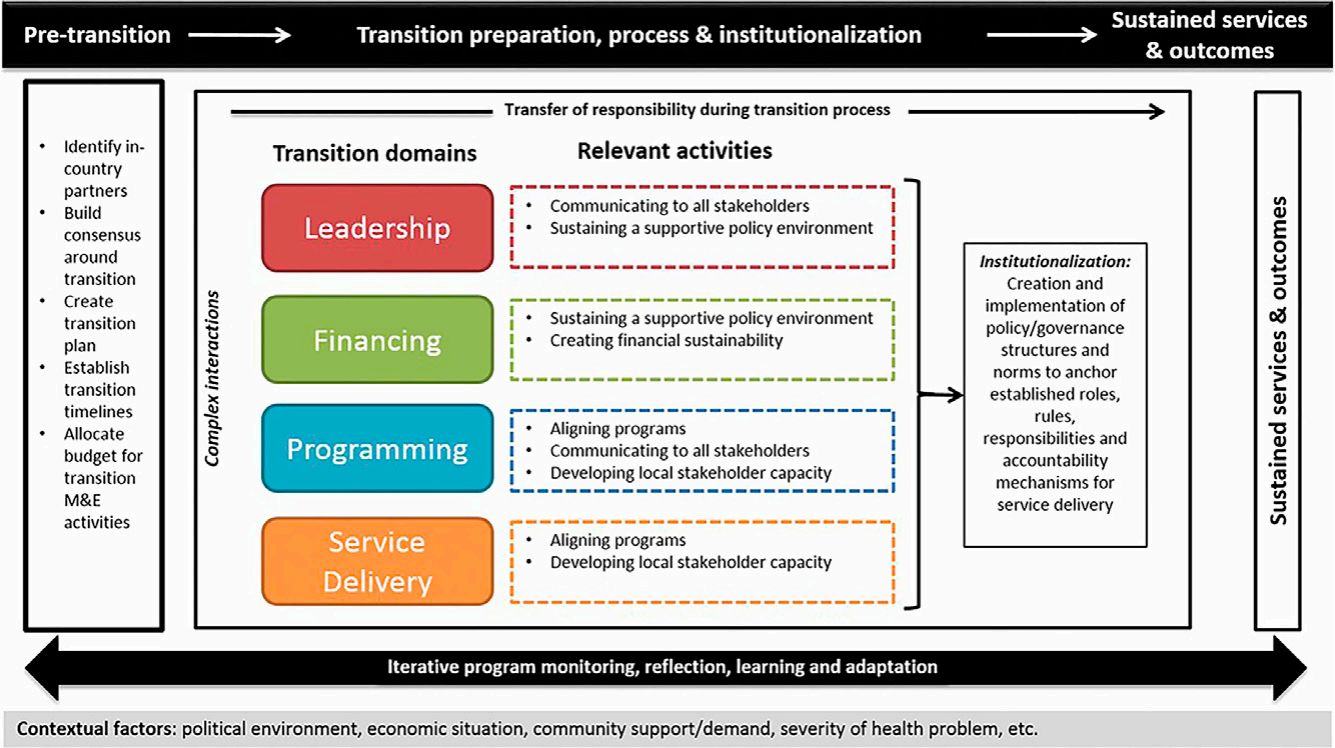
Conceptual framework for donor transitions (from Bao et al. 2015^17^).

Bao et al.^17^ proposed a conceptual framework for monitoring and evaluation of donor transition (Figure 1) that identifies four key domains where responsibility shifts over time from the donor to the recipient during transition:

- *Leadership*: Ownership, political will, and commitment among local stakeholders to take over responsibility for program implementation. Highly context specific, often requires involvement from actors outside the health sector (e.g., Ministry of Finance).
- *Financing*: Financial support for continued implementation; likely to be secured from multiple sources and may require lobbying.
- *Programming*: Program management and operational responsibilities, including workforce, regulatory, and administrative activities.
- *Service delivery*: Responsibility for logistics of service delivery, including human resources, procurement, etc. Applicable in cases where donors have been directly delivering services.

The authors contrast several transition types depending on how many of the key domains are involved in the program under transition. Although all transition types require investment in the leadership domain, engagement with other domains varies by transition type, with more domains resulting in more complexity (see Box 1).

Box 1 **Transition types, adapted from Bao et al. 2015^17^**

**Table:** 

**Transition type A (e.g., GAVI in Eastern Europe)** The core component to be transitioned is funding because program management and service delivery were already the responsibility of local partners.	↓Increasing complexity
**Transition type B (e.g., family planning in Latin America)** Both funding and program management are transitioned, whereas service delivery remains in the hands of local partners.	
**Transition type C (e.g., Avahan in India)** Transfer of responsibilities includes funding, program management, and service delivery from external partners (e.g., funders and implementing agencies) to local entities.	

However, as donor transitions become more common, programs may shift between transition types before fully transitioning away from external support. In this paper, project staff and implementers describe the transition process of a program in Mozambique as its funding shifted from one external partner to another while giving local counterparts more control over technical and managerial implementation. We also reflect on facilitating factors, challenges, and lessons learned to inform programs undergoing similar experiences in the future.

## COUNTRYWIDE MORTALITY SURVEILLANCE FOR ACTION – MOZAMBIQUE

The Countrywide Mortality Surveillance for Action (COMSA) – Mozambique program was established in 2017 with the goal of establishing a national, community-based surveillance system for mortality and causes of death (Box 2). The overall design relies on community-based surveillance assistants to conduct regular data collection on vital events at the community level, supplemented with verbal and social autopsies conducted by teams deployed from the provincial level. COMSA also liaised with the Children’s Health and Mortality Prevention Surveillance site in Zambezia province to refine the calibration of the algorithms used to estimate cause-of-death results for under-five children.

Box 2 **Key features of Countrywide Mortality Surveillance for Action – Mozambique**

**Table:** 

**Sample:** Representative at national and provincial levels.
**Coverage:** 700 randomly selected clusters for a total of 180,000 households and over 800,000 population (out of total 29 million Mozambique population).
**Staff:** 700 community surveillance assistants; 48 verbal and social autopsy data collectors; 22 provincial-level managers, including Maputo City; 12 central-level technical staff; additional administrative and management support.
**Main data generated:** Birth and death rates by age, sex, location; causes of death, social determinants of deaths.

COMSA was developed by the Institute for International Programs at Johns Hopkins University (JHU) in partnership with the Instituto Nacional de Saúde (National Institute of Health [INS]) and the Instituto Nacional de Estatística (National Institute of Statistics [INE]) in Mozambique, with funding from the Bill and Melinda Gates Foundation. JHU provided technical assistance, and INS and INE were the primary implementers in-country (Table 1). The Gates Foundation established the grant with JHU, with a subcontract to INS. A memorandum of understanding guided the collaboration between INS and INE, with INE overseeing data-collection operations nationwide. A national advisory group was established to advise on strategic, programmatic, methodological, and financial aspects of COMSA and included high-level representation from INS, INE, Ministry of Health (MISAU), Ministry of Justice (MJCR), CDC, United Nations Children’s Fund (UNICEF), U.S. Agency for International Development (USAID), and WHO.

**Table 1:** Division of responsibilities within COMSA

Institution	Characteristics	Role within COMSA
INE (autonomous official statistics organization, affiliated with the Ministry of Finance)	Conducts surveys and census regularly Provincial-level infrastructure, including staff, vehicles, and other resources Responsible for contributing to vital statistics	Coordination and supervision of data collection at central, provincial, and community level Data management through regular and contractual staff
INS (autonomous parastatal organization accountable to the MISAU)	Responsible for generating health information (surveys, surveillance, and research) Limited subnational footprint Administrative and financial management structures suitable for project management	Administrative and financial management of project funds (with INE as subgrantee) Technical operations for cause of death data, overall data management and supervision Liaison to CHAMPS Dissemination of results and liaison to MISAU for data use
JHU	Extensive demography, statistics, and evaluation expertise	Primary recipient of funding (with INS as subgrantee) Technical assistance for implementation, scale-up and supervision Development of cause of death modeling, and calibration of cause-of-death models Established online platforms for data collection, analysis, and dissemination

CHAMPS = Children’s Health and Mortality Prevention Surveillance; COMSA = Countrywide Mortality Surveillance for Action; INE = National Institute of Statistics; INS = Instituto Nacional de Saúde (National Institute of Health); JHU = Johns Hopkins University; MISAU = Ministry of Health.

Although COMSA was designed as a demonstration project, the Gates Foundation and the implementing partnership hoped that COMSA could be institutionalized for longer-term benefits. In June 2019, 15 months after implementation began, the Gates Foundation requested that JHU develop a transition implementation plan to support the transfer of all COMSA responsibilities to INS, INE, and other partners.

## TRANSITION IMPLEMENTATION

From the outset, it is important to highlight Mozambique’s status as a low-income country. In 2020, Mozambique spent only 7.6% of its gross domestic product (GDP) on health, thus making it heavily reliant on outside donors, with over 50% current health expenditures coming from external assistance.^18^ From a workforce standpoint, Mozambique has limited numbers of specialized staff, such as epidemiologists, demographers, statisticians, programmers, etc., all of which are necessary to lead the development and implementation of a new community-based surveillance system like COMSA.

**Figure 2.**
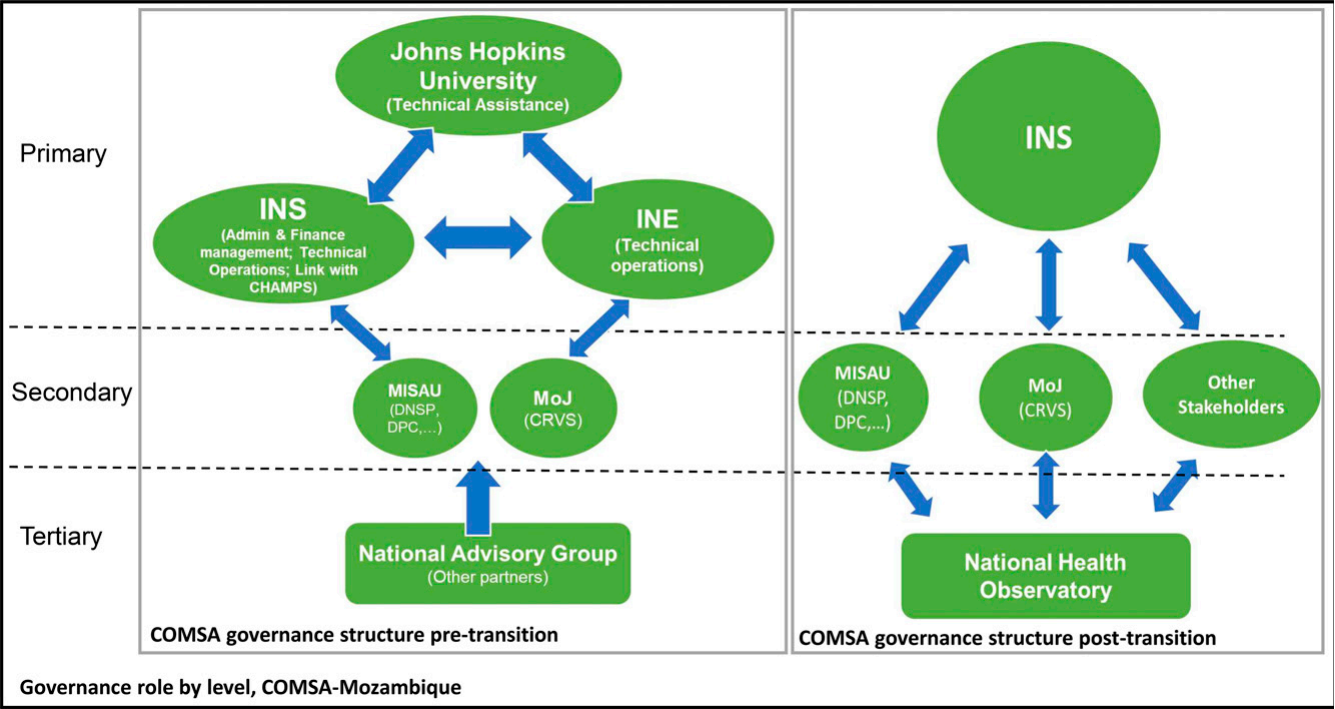
Shift in Countrywide Mortality Surveillance for Action – Mozambique governance during transition. CHAMPS = Children’s Health and Mortality Prevention Surveillance; CRVS = Civil Registration and Vital Statistics; DNSP = National Public Health Directorate; DPC = Directorate for Planning and Cooperation; INE = National Institute of Statistics; INS = National Institute of Health; MISAU = Ministry of Health; MoJ = Ministry of Justice and Constitutional and Religious Matters.

The specifics of the COMSA system and results are described elsewhere.^19^ From a transition domains standpoint, leadership of COMSA was shared by all three institutions, with JHU offering technical expertise and INS/INE providing implementation know-how; program management and operations were likewise shared responsibilities (Figure 2). JHU held the primary responsibility for financing and design as the primary recipient for funding, whereas INS and INE held the primary responsibility for implementation. Because INS and INE were already responsible for implementation of activities on the ground, COMSA fit a Type B transition, which includes domains of leadership, financing, and program management. We begin by briefly describing the transition process, followed by reflections by each domain type.

## TRANSITION PROCESS

By August 2019, a dedicated transition technical working group (TTWG) was established, constituted by key implementing staff from JHU, INS, and INE (see Table 2 for timeline). The TTWG was responsible for technical and operational transition planning, including transition implementation plans, pursuing future funding, and monitoring progress. The initial intention was to have shared leadership of the TTWG between INS and INE; however, over time—and in part due to internal staffing changes at each agency—the INS lead on the TTWG became the key decision-maker, with the JHU staff trying to ensure regular attention to transition issues.

**Table 2:** COMSA transition timeline

2017	January	COMSA project planning phase begins
2018	March	Data collection begins
2019	April	Gates Foundation requests transition planning
	July	Transition planning approach discussed with local partners
	August	Transition TWG established (then meets regularly)
	December	Transition implementation plan prepared by TTWG agreed by INS and INE
2020	January	Transfer of power after 2019 national elections
	February	TTWG begins engagement with MISAU
	March	First COVID-19 case reported in Mozambique
	June	Global Fund application submitted
	October	Global Fund application approved in principle
	December	JHU closes out implementation role (planned)
	December	INE casts doubt on their post-transition role
2021	March–June	Rapid evaluation of COMSA implementation
	May	Global Fund funding available for use; contracting delays continue
	June	JHU closes out financial support; implementation support and technical assistance continue
	August	INE finalizes its withdrawal from future implementation
	December	JHU implementation support role ends (actual)
2022	April (expected)	JHU technical assistance role ends
	December (actual)	JHU technical assistance role ends

COMSA = Countrywide Mortality Surveillance for Action; INE = National Institute of Statistics; INS = National Institute of Health; JHU = Johns Hopkins University; MISAU = Ministry of Health; TWG = technical working group; TTWG = transition technical working group.

**Figure 3.**
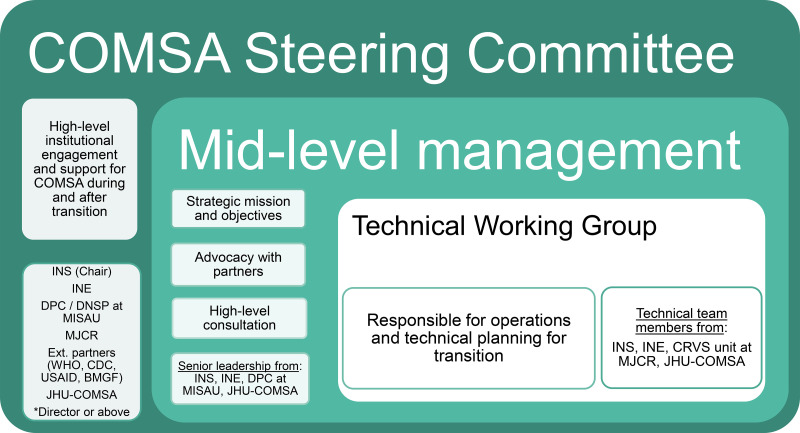
Countrywide Mortality Surveillance for Action (COMSA) transition governance structure. BMGF = Bill & Melinda Gates Foundation; CRVS = Civil Registration and Vital Statistics; DNSP = National Public Health Directorate; DPC = Directorate for Planning and Cooperation; INE = National Institute of Statistics; INS = National Institute of Health; JHU = Johns Hopkins University; MISAU = Ministry of Health; MJCR = Ministry of Justice; USAID = U.S. Agency for International Development.

The TTWG was intended to be nested within a larger governance structure that would provide strategic guidance, advocate with external partners, and support higher-level commitment to institutionalization post-transition (Figure 3). However, the relationship with the mid-level management group was irregular, and, due to the COVID-19 pandemic, the TTWG never engaged with the Steering Committee.

A transition-specific budget and two dedicated, part-time transition staff were hired (with additional Gates Foundation funding) to shepherd the transition process because it was determined that current COMSA staff would be unable to dedicate the necessary time and attention to keeping the transition progressing while still meeting their regular COMSA duties. In addition to offering persistent momentum, these staff conducted transition-specific activities, such as budget projections for future implementation. One staff member was based in Baltimore and liaised directly with the JHU team, and the other was in Mozambique and liaised with INS, INE, and other local stakeholders; both were members of the TTWG.

By the end of 2019, the TTWG established a transition implementation timeline outlining activities related to each of the key domains described below. The initial endpoint for the timeline was December 2020, allowing 1 year for transition implementation. However, the COVID-19 pandemic disrupted country-level activities and extended COMSA’s overall project timeline. COMSA’s platform was partially repurposed to support the pandemic response,^20^ and several key COMSA actors (e.g., INS local lead) were tasked with leading other COVID-19 activities. In general, it was difficult to maintain attention and progress on transition planning against so many competing issues, until critical deadlines arrived. The project’s implementation support ended by December 2021, with continued technical assistance from JHU for an additional 4 months.

## TRANSITION DOMAIN – LEADERSHIP

The TTWG’s work under the Leadership domain was focused on ensuring broader stakeholder commitment to sustaining COMSA activities post-transition and establishing an appropriate post-transition governance plan. Stakeholder engagement and advocacy took place internally within INS and INE, and externally with the MISAU and others, to broaden the involvement of key Mozambican actors. INS and INE signaled commitment and buy-in from the start because they felt that the program was valuable, established a platform for continuous community-based surveillance that could be useful beyond the initial COMSA focus, and offered critical evidence necessary for national program and policy planning.

The TTWG’s engagement with MISAU was focused on integrating COMSA as a program within the Ministry’s information system portfolio. Up to this point, MISAU had viewed COMSA as donor-funded demonstration project. Their engagement had been limited but with significant interest in seeing what evidence the system could generate. To broaden interest and support, the TTWG intended to consult with different departments within MISAU (e.g., maternal and child health, immunization, etc.) to develop a list of their information needs that could eventually be served by COMSA’s community-based surveillance system. However, planned engagement activities were delayed by post-election transition of power and staff turnover in early 2020, and then the arrival of COVID-19 made these consultations impossible because MISAU’s attention was focused on pandemic response. Likewise, the TTWG’s intention to bring the MJCR Civil Registration and Vital Statistics (CRVS) unit into COMSA’s post-transition management team was unsuccessful.

Strategic planning under this domain included developing a structure that reflected a more locally led approach to the system, including a new name and governance structure. Stakeholders felt a new name with Portuguese words was critical for local ownership, rather than the English words from COMSA. The new name agreed upon by the TTWG and the mid-level management group in December 2019 was Sistema Comunitário de Vigilância em Saúde e de Eventos Vitais (SIS-COVE). Initially the post-transition governance structure was modeled after the existing structure under COMSA but dividing JHU’s responsibilities between INS and INE. Day-to-day responsibilities would lie with a technical coordination team, featuring key members of all Mozambican partners, that would answer to a consultative committee that included senior members of partners as well as representation from SIS-COVE funders. SIS-COVE was intended to be institutionalized under the Surveys and Health Observatory Directorate at INS and to remain under the Demographic, Vital and Social Statistics Directorate at INE. However, the joint governance structure was never agreed upon or finalized due to INE’s concern regarding the alignment on COMSA/SIS-COVE objectives and INE’s mission (see below).

At the end of 2019, during a high-level meeting between directors from INS and INE, INE’s leadership began to question its involvement in COMSA post-transition, which threw final planning and decision-making into disarray. INE leadership expressed concern about the burden COMSA activities placed on its staff and provincial offices while not being sufficiently aligned with INE’s mission. A rapid evaluation of COMSA’s implementation was requested by INE before they would finalize their decision on future involvement; the rapid evaluation was completed by June 2021 with results presented to all partners. After reviewing the evaluation results and further discussion, INE decided step away from day-to-day implementation activities and instead empower INS to fulfill the statistics role for the health sector with INE continuing to provide technical assistance, as needed. To that end, INE designated INS as part of the official national statistics system as a delegated institution (known as ODINE) to continue SIS-COVE implementation. In its role as main implementer, INS then designed a governance structure that would support the continuous management necessary for SIS-COVE’s operations.

## TRANSITION DOMAIN – FINANCING

Concerns about financing for SIS-COVE post-transition were widespread among COMSA’s partners, including the Gates Foundation, and became a significant focus of the transition-planning work. Expenditure and financial projections for 1- and 3-year timeframes were developed to inform budget requests presented to stakeholders and submitted to other funding agencies. The initial hope had been that domestic resources could be mobilized to finance SIS-COVE, but as other external funding opportunities arose, the goal became that the Gates Foundation funding for COMSA activities channeled via JHU would shift seamlessly to other funders supporting INS and INE directly to deliver SIS-COVE.

The TTWG was able to pursue financial commitment from different donors via their pre-existing relationships. For example, INE incorporated requests for SIS-COVE implementation funds into a proposal submitted to the World Bank, intended to support INE’s work on strengthening the country’s CRVS. INS, through its relationship with MISAU, submitted SIS-COVE for funding consideration into Mozambique’s 2020 Global Fund application and was successfully funded this way for 3 years. Notably, these two funding opportunities benefited from fortuitous timing because the funding cycles coincided with SIS-COVE’s transition planning.

An important consideration for SIS-COVE is that disease-specific funding mechanisms are not well suited to support a broad health surveillance program with continuous data collection. Restrictions on contractual staff and field logistics (e.g., vehicles for data collection visits) led to cumbersome bureaucratic difficulties, which were in stark contrast with the flexible funding offered under the Gates Foundation. The intended window whereby the Gates Foundation–JHU contract to INS would end and new funders would step in was January 2021; however, delays in the contracting with the Global Fund resulted in JHU extending its contract with INS to cover operational expenses through April 2021 then shifting to only technical assistance. Once the Global Fund financing was allocated in May 2021, protracted negotiations between INS, the Global Fund, and its representatives over how to continue pre-existing staff contracts led to staff salaries being delayed by 5 months or more and severely affected SIS-COVE operations.

## TRANSITION DOMAIN – PROGRAM MANAGEMENT

To support the program management aspect of transition, the TTWG began by documenting all operational activities, identifying where core responsibilities lay (e.g., JHU, INS, INE), and developing a plan for transfer of JHU responsibilities to relevant INS and INE staff. Timelines were identified for transferring responsibilities, and the TTWG also attempted to identify critical capacity needs that JHU could try to address prior to transition. COVID-19 disrupted training plans by both curtailing planned in-person capacity-building workshops and diverting COMSA staff into pandemic response. Workshops were delivered virtually in late 2020; however, it was recognized that these were not sufficient, and refresher trainings were delivered in late 2021. The TTWG also supported INS as it restructured its governance approach to SIS-COVE post-transition.

Johns Hopkins University held COMSA’s contracts for the servers used for data collection, analysis, and public dissemination. Thus, the transfer of information technology (IT) was an essential aspect of transition that was more complex than anticipated, so the TTWG established a sub-group focusing strictly on IT aspects given the technical complexity involved. When COMSA was established, the technical requirements for data servers could not be met by Mozambican providers, so the contracts had been established with external companies. Given legal restrictions around arrangements of this nature, the IT group had to explore whether local, government-managed servers could be used to house SIS-COVE data post-transition. This proved to be difficult and time-consuming, but eventually, high-availability servers were identified at INS that could house SIS-COVE data post-transition.

A more substantial issue arose around human resources for IT and data management. The JHU-developed technology used for the data analysis server could not be easily transferred to or learned by Mozambican partners. Despite training efforts between JHU and local COMSA IT staff, some gaps could not be overcome, such as learning a new computer programming language in a short time. The JHU team worked to automate as many processes as possible to facilitate operations. Further, and with implications for sustainability, the most highly qualified IT staff working on COMSA (and later SIS-COVE) are contractual employees, not regular INS or INE staff. Because of their less permanent status, contractual staff had less authority in terms of guiding or making recommendations for transition, making it more challenging for them to lead the IT transition. In the end, some of the IT staff were retained on contract post-transition and continue to provide operational support and institutional memory.

## TRANSITION DOMAIN – SERVICE DELIVERY

One critical facilitator for the transition to SIS-COVE is that the core responsibilities for managing and implementing local COMSA activities were already fully integrated into INS and INE structures. For example, supervisory structures for local staff relied on INE’s provincial staff and vehicles, and human resources contracts were managed through INS’s systems. Although at the outset we did not anticipate that the service delivery domain would apply here, INE’s decision to discontinue its involvement in SIS-COVE’s daily operations meant that INS had to reconfigure implementation arrangements to be entirely within its remit. This meant establishing a completely internal management structure and developing a system for supporting local-level data collection because COMSA had been reliant on INE’s substantial provincial-level footprint, which INS does not have yet. This eventuality arose late and unexpectedly in transition planning and took many months to resolve, resulting in considerable uncertainty during the transition process.

Table 3 summarizes the concrete activities that the TTWG pursued in support of transition.

**Table 3:** TTWG activities by transition domain

Leadership	Advocacy and engagement with other government agencies Engage with mid-level management group for strategic decision-making (e.g., new name)
Financing	Advocacy and engagement with funders Develop budget projections for future funding Support applications for funding to development partners Negotiations with funders, as needed
Program management	Review of COMSA activities and decisions on what to transition or eliminate Engage with internal teams at JHU, INS, and INE to prepare and execute transfer of responsibilities Establish informational technology sub-TWG Capacity building needs assessment, and capacity building activities
Service delivery	Develop proposal for post-transition governance of COMSA activities

COMSA = Countrywide Mortality Surveillance for Action; INE = National Institute of Statistics; INS = National Institute of Health; JHU = Johns Hopkins University; TWG = technical working group; TTWG = transition technical working group.

## FACILITATORS AND CHALLENGES

Table 4 captures the facilitators and challenges for SIS-COVE’s transition. It is important to note that many of these factors were heavily influenced by timing and chance.

**Table 4:** Facilitators and challenges to SIS-COVE transition planning

Facilitators	The COMSA project model already embedded significant implementation and management responsibility with local agencies. Despite the initial 1-year timeline, transition was helped by COMSA being designed within local structures from the outset. High-level commitment from core implementers (INE and INS) at the outset meant that advocacy work could focus on stakeholders external to COMSA. Establishment of dedicated personnel and budget to support transition. Fortuitous timing for pursuing other donor financing. COMSA’s value as a potential community-based surveillance platform during the COVID-19 response raised its profile and increased interest from funders.
Challenges	Gaining buy-in from other government agencies (e.g., government ministries), and eventually retaining operational involvement from INE. Transferring IT capabilities and management to local partners; this was one area where JHU and contractors held most of the technical capacity. Navigating tensions between INS and INE around future roles and responsibilities, such as reviewing existing implementation structures and making necessary changes to human resources or accountability structures. COVID-19 response drew away attention and energy from transition activities until crucial deadlines arrived: all planned activities were delayed, staff attention diverted, difficult for JHU team to support from afar. Bridging the gap between SIS-COVE’s operational activities and the strict management bureaucracy under Global Fund financing leading to substantial delays in activities, which threaten the success of the program. Last-minute negotiations with key stakeholders, such as INE’s continued involvement and Global Fund contracting, generated substantial uncertainty making it difficult to finalize plans and complete transition activities.

COMSA = Countrywide Mortality Surveillance for Action; INE = National Institute of Statistics; INS = National Institute of Health; IT = information technology; JHU = Johns Hopkins University; SIS-COVE = Sistema Comunitário de Vigilância em Saúde e de Eventos Vitais.

## DISCUSSION

COMSA was designed from the outset to address local capacity needs and to be embedded with local institutions so that the program had a secure footing toward sustainability. However, planning for the sustainability of a surveillance program turned out to be different from planning for transition. All in all, we underestimated the transition effort, and our experience generates several lessons for others pursuing similar transfers of responsibility.

First, despite a program structure that relied heavily on existing local implementers, there were aspects that proved to be challenging in shifting from COMSA to SIS-COVE. For example, the distribution of analytical and IT responsibilities was unbalanced between JHU and local partners. In part, this reflected the strategic advantages offered by each partner at the outset of the program but also resulted in a system where both technological and human resources could not be quickly replicated in Mozambique to the capabilities present within JHU. Likewise, the engagement with other local stakeholders proved to be difficult. INE and INS have pre-existing relationships with other ministries and agencies that could have been leveraged sooner to offer a stronger argument for institutionalization. However, COMSA’s transition was a catalyst to INS becoming an ODINE, which speaks to broader structural changes for health information systems locally.

Second, a fair amount of pragmatism is necessary when planning a transition of this nature. As noted elsewhere, program activities are rarely retained in the same form post-transition as before,^21^ and funding sources are not necessarily available in the amount and nature that funders and implementers prefer. The fortuitous timing of open funding applications made it possible to secure medium-term funding for SIS-COVE, but this is not necessarily a replicable outcome.

Third, JHU and its partners felt pressure to deliver on their grant obligations to the Gates Foundation, so some of the programmatic choices made at the outset of COMSA functioned as operational or bureaucratic shortcuts necessary to aid implementation but were difficult to correct for during transition, such as data server contracts held by JHU. Further, the initially short timeline for transition implementation (∼12 months), later complicated by COVID-19, made it difficult and time consuming to address these gaps. It is likely that if transition had been the intention from the early of days COMSA (rather than midway through), then JHU, INS, and INE teams would have made different choices that strengthened the basis for institutionalization.

COMSA experienced a mixed type of transition whereby the leadership, management, and delivery roles were fully transferred to local country counterparts but financing for SIS-COVE continues to rely on development partners. Importantly, continuing with external funding resulted in trade-offs. From an operational standpoint, under the Global Fund’s structures, INS has less flexibility and autonomy paired with rigid oversight and accountability measures that are not well suited to health systems investments like these. In the long run, shifting between development partners leaves SIS-COVE vulnerable to continuously seeking financing.

Our experience and the ensuing post-transition plans for COMSA raise important questions about the obligations of development partners when it comes to systems investments and transitioning programs. COMSA is a notable investment in surveillance system innovation—the kind of health systems strengthening activities we hope to see more—yet, it started as a short-term demonstration project. Despite buy-in from multiple national agencies committed to improving health data, COMSA still fell victim to the typical short-term funding cycle demands of donors, which not only rushed the transition process but also meant competing for implementers’ attention.

## CONCLUSION

We believe that donor transitions like these will continue in Mozambique and elsewhere. As such, donor-funded programs should use eventual transition as a critical endpoint during design, even if transition has not been predefined at the outset; this requirement should come from funders, implementers, and governments. Likewise, assessments of transition readiness could help inform the timing of transition and establish a stakeholder management plan to be incorporated into transition planning. Also, transition processes require specific attention, investment, and time to minimize negative consequences on program objectives. We echo the repeated calls for systems investments with a longer runway to ensure adaptation over time and eventual success, which applies to all forms of development partners. More broadly, it is critical to acknowledge that mixed transitions—where health programs become locally owned and operated while relying on external funding—will become more common, which undermines sustainability and perpetuates unbalanced power dynamics.

## Financial Disclosure

Financial support: The COMSA Mozambique project and its transition efforts were funded by the Bill and Melinda Gates Foundation (Grant no. OPP1163221). The views expressed in this report do not necessarily reflect the views of the Bill & Melinda Gates Foundation.
